# A whey protein-based multi-ingredient nutritional supplement stimulates gains in lean body mass and strength in healthy older men: A randomized controlled trial

**DOI:** 10.1371/journal.pone.0181387

**Published:** 2017-07-18

**Authors:** Kirsten E. Bell, Tim Snijders, Michael Zulyniak, Dinesh Kumbhare, Gianni Parise, Adrian Chabowski, Stuart M. Phillips

**Affiliations:** 1 Exercise Metabolism Research Group, Department of Kinesiology, McMaster University, Hamilton, ON, Canada; 2 NUTRIM, Department of Human Biology and Movement Sciences, Maastricht University Medical Center, Maastricht, The Netherlands; 3 Department of Medicine, McMaster University, Hamilton, ON, Canada; 4 Department of Physiology, Medical University of Bialystok, Bialystok, Poland; University of Alabama at Birmingham, UNITED STATES

## Abstract

Protein and other compounds can exert anabolic effects on skeletal muscle, particularly in conjunction with exercise. The objective of this study was to evaluate the efficacy of twice daily consumption of a protein-based, multi-ingredient nutritional supplement to increase strength and lean mass independent of, and in combination with, exercise in healthy older men. Forty-nine healthy older men (age: 73 ± 1 years [mean ± SEM]; BMI: 28.5 ± 1.5 kg/m^2^) were randomly allocated to 20 weeks of twice daily consumption of either a nutritional supplement (SUPP; n = 25; 30 g whey protein, 2.5 g creatine, 500 IU vitamin D, 400 mg calcium, and 1500 mg n-3 PUFA with 700 mg as eicosapentanoic acid and 445 mg as docosahexanoic acid); or a control (n = 24; CON; 22 g of maltodextrin). The study had two phases. Phase 1 was 6 weeks of SUPP or CON alone. Phase 2 was a 12 week continuation of the SUPP/CON but in combination with exercise: SUPP + EX or CON + EX. Isotonic strength (one repetition maximum [1RM]) and lean body mass (LBM) were the primary outcomes. In Phase 1 only the SUPP group gained strength (Σ1RM, SUPP: +14 ± 4 kg, CON: +3 ± 2 kg, *P* < 0.001) and lean mass (LBM, +1.2 ± 0.3 kg, CON: -0.1 ± 0.2 kg, *P* < 0.001). Although both groups gained strength during Phase 2, upon completion of the study upper body strength was greater in the SUPP group compared to the CON group (Σ upper body 1RM: 119 ± 4 vs. 109 ± 5 kg, *P* = 0.039). We conclude that twice daily consumption of a multi-ingredient nutritional supplement increased muscle strength and lean mass in older men. Increases in strength were enhanced further with exercise training.

***Trial Registration*:** ClinicalTrials.gov NCT02281331

## Introduction

Age-related declines in muscle mass and function, termed sarcopenia, contribute to various negative health outcomes: metabolic disorders like type 2 diabetes mellitus and progression to frailty [1]. Muscular strength is a strong and independent predictor of all-cause mortality in older adults [2]; hence, solutions to attenuate sarcopenic declines are imperative [3]. Resistance exercise training (RET), particularly when combined with nutritional supplements such as protein [4] and creatine [5], is an effective strategy to counter muscle mass and strength loss. In older adults, RET has also been shown to induce modest yet significant reductions in cardiovascular disease risk [6], improvements in metabolic health [7] and aerobic capacity [8], and reduce fall risk [9].

The combination of RET with protein and creatine supplementation is a potent stimulus for increases in strength and lean mass. Recent studies also suggest that supplementation with vitamin D [10] and omega-3 polyunsaturated fatty acids (n-3 PUFA) [11] may also be effective in augmenting strength and hypertrophy. In addition to RET, evidence that high-intensity interval training (HIIT) may stimulate muscle protein synthesis [12], improve glycemic regulation [13], and increase aerobic capacity in older persons [14] is accumulating. Few studies have employed a multicomponent supplement with a combination of exercise modalities in older populations [15, 16]. However, such an approach may be prudent because individual variability in response to nutritional supplementation, RET, and HIIT, is significant. Therefore, a multicomponent nutritional strategy, especially when combined with multimodal exercise training, would be more likely to yield a positive outcome in a greater proportion of older persons compared to isolated supplements.

The primary objective of this trial was to determine whether twice daily consumption of a supplement containing whey protein, creatine, calcium, vitamin D, and n-3 PUFA (rich in eicosapentanoic acid [EPA] and docosahexanoic acid [DHA]) could stimulate gains in strength, physical function, lean body mass, and metabolic health in a group of healthy older men following 6 weeks of supplementation. We also determined if the addition of exercise would potentiate supplement-mediated gains in strength, physical function, lean body mass, and metabolic health following a 12 week combined RET + HIIT exercise program. We hypothesized that our multi-ingredient supplement would induce improvements in these outcomes independent of exercise and that we would observe an additive effect of the supplement when combined with exercise training.

## Methods

### Screening and recruitment

Forty-nine health older men participated in this randomized, double-blind, placebo-controlled parallel group trial which took place between December 2014 and September 2016 (see S1 File. Study Protocol). Potential participants were screened first by telephone to ensure they were non-smokers ≥ 60 years old, had a body mass index (BMI) in the normal-overweight range (between 18.5 and 30.0 kg/m^2^), and had normal resting blood pressure or stage I hypertension (systolic blood pressure [BP] ≤ 140–159 mmHg; diastolic BP ≤ 90–99 mmHg). In the previous 6 months, potential participants had not participated in any structured resistance or aerobic exercise training program. Exclusion criteria included: significant weight loss or gain in the past 6 months; regular use of: non-steroidal anti-inflammatory drugs, simvastatin, atorvastatin, or anticoagulants; injuries preventing safe participation in an exercise program; diabetes mellitus; cancer; infectious disease; unstable cardiac; and/or gastrointestinal disease.

**Fig 1:**
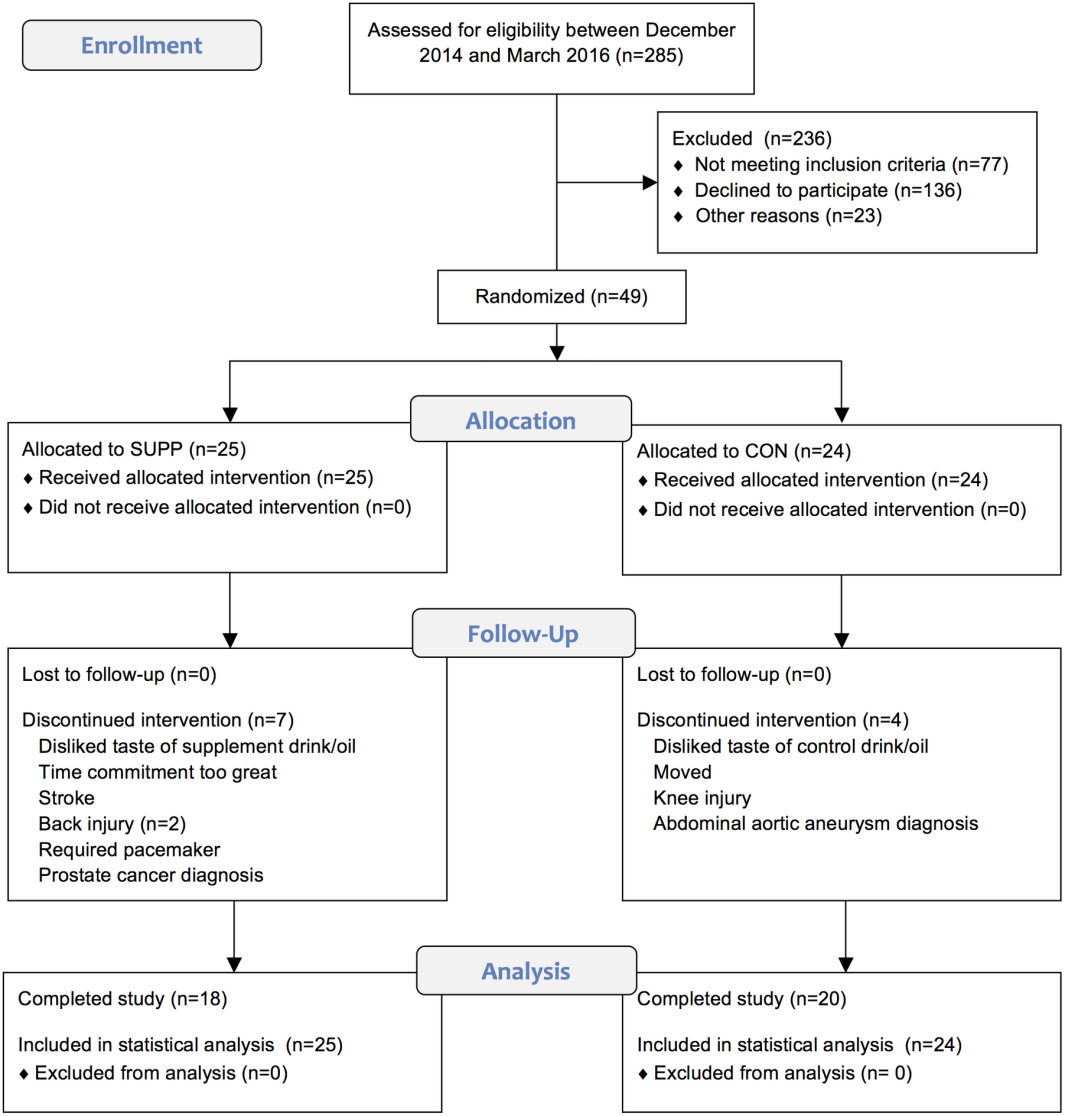
CONSORT flow diagram illustrating the movement of participants through the study, which was conducted between December 2014 and September 2016 (see S2 File. CONSORT Checklist).

To confirm eligibility, subjects were required to be non-diabetic, based on an oral glucose tolerance test (OGTT; fasting blood glucose < 7.0 mM; 2 h blood glucose < 11.1 mM), complete a physical activity readiness questionnaire (PAR-Q) [17], and demonstrate normal cardiac function during a maximal exercise stress test on a cycle ergometer. This trial was approved by the Hamilton Integrated Research Ethics Board and complied with the guidelines set out in the Canadian Tri-Council policy statement on ethical conduct for research involving humans (http://www.pre.ethics.gc.ca/pdf/eng/tcps2/TCPS_2_FINAL_Web.pdf). All participants were informed of the nature and possible risks of the experimental procedures before their written informed consent was obtained. This study ended in September 2016 upon completion of post testing by the last group of participants to begin the protocol. Full details concerning the flow of participants through this study can be found in Fig 1.

Sample sizes were calculated based on detection of increases in leg press isotonic strength of 3.25 kg (standard deviation: 1.5 kg) observed during creatine supplementation combined with RET in older persons compared to RET alone or with a placebo [5]. Assuming similar response variance in our subjects during a 2-way repeated measures ANOVA and setting power to 80% with alpha at 0.05 (2-tailed) yielded an estimate of 19 subjects per group. To be conservative and allow for a potential 20% dropout rate we aimed for 25 subjects per group.

### Experimental design

**Fig 2:**
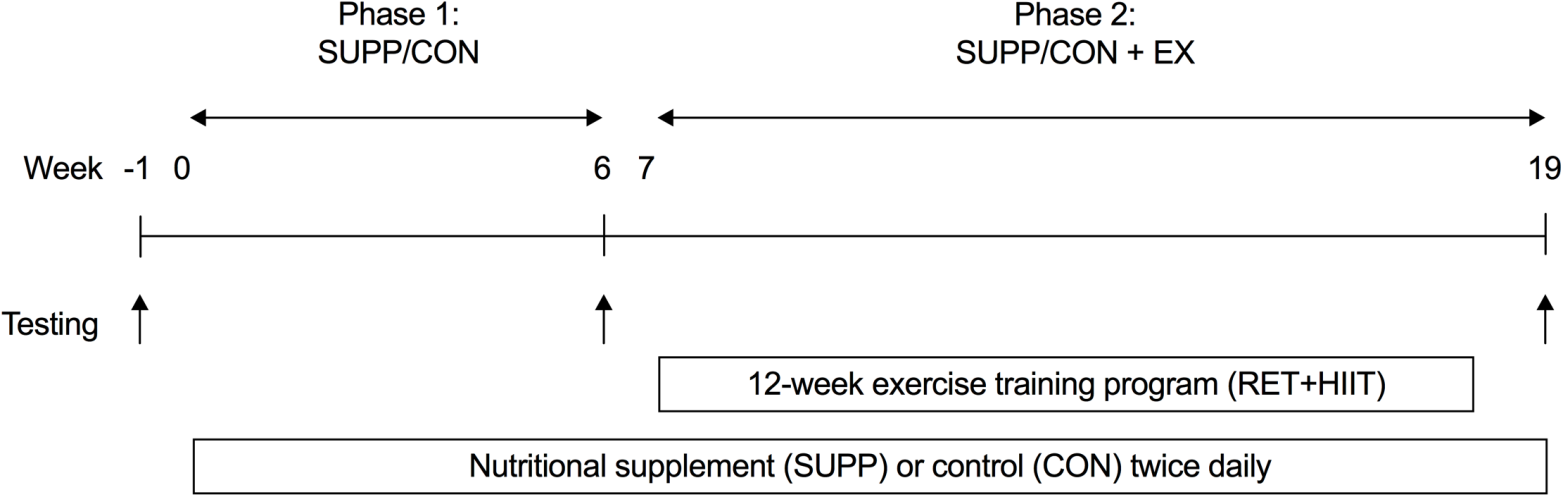
Schematic of study design. Participants consumed either a multi-ingredient protein-based nutritional supplement (SUPP) or control (CON) drink for 20 weeks total (from weeks 0–19, inclusive), and completed a 12 week exercise training program (RET twice per week and HIIT once per week) between weeks 7–18. Testing occurred at weeks -1 (baseline), 6, and 19, and included the following assessments: isotonic strength (1RM), aerobic fitness (VO_2_peak), physical function, body composition (DXA), and a 75g OGTT. Phase 1: SUPP/CON took place between weeks 0–6, and Phase 2: SUPP/CON + EX took place between weeks 7–19. 1RM, one repetition maximum; DXA, dual-energy x-ray absorptiometry; HIIT, high-intensity interval training; OGTT, oral glucose tolerance test; RET, resistance exercise training; VO_2_peak, peak oxygen uptake.

Eligible subjects were allocated to receive either a multi-ingredient nutritional supplement (SUPP) or a control (CON) drink for 20 weeks (Fig 2). We employed a coded (group A versus group B) block randomization scheme (block size: 10 participants) generated using http://www.randomization.com/ to sequentially allocate subjects to groups in order of enrolment. A key to the randomization code was held by an investigator who was not directly involved with subject recruitment, training, or testing. Subjects, as well as investigators who were responsible for recruiting, training, and/or testing subjects, were blind to the individual group assignments.

After 6 weeks of consuming their study beverages (Phase 1: SUPP or CON), subjects completed a 12 week supervised exercise training program while continuing to consume their assigned beverages (Phase 2: SUPP + EX or CON + EX). Testing occurred at weeks -1 (pre-intervention), 6, and 19. The following assessments were conducted over the course of each testing week: isotonic strength (one repetition maximum [1RM]), aerobic fitness (peak oxygen uptake [VO_2_peak]), physical function, body composition by dual-energy x-ray absorptiometry (DXA), OGTT, dietary intake (3-day food records), and habitual physical activity (accelerometer).

### Nutritional supplements

The experimental supplement was composed of whey protein, creatine, calcium, vitamin D, and n-3 PUFA. All ingredients except for the n-3 PUFA (and control oil) were packaged in powder form in individual sachets (Infinit Nutrition, Windsor, ON). Subjects prepared the beverages at home by mixing the contents of 1 sachet with 425 mL water and consumed two drinks daily: 1 h after breakfast, and again 1h prior to retiring to bed. Subjects measured out and consumed 10 mL of oil once per day (the SUPP oil contained 3000 mg n-3 PUFA with 1400 mg eicosapentanoic acid [EPA] and 890 mg docosahexaenoic acid [DHA]; CON was safflower oil) with their morning study beverage. The powder and oil provided to the CON group were matched in flavor and odour to the active forms. The exact composition of and nutrition information for the supplement and control drinks and oils can be found in Table 1. All study beverages and oils were labelled in a blinded manner (Infinit Nutrition, Windsor, ON). Participants were instructed not to alter their habitual dietary or physical activity habits for the duration of the study.

**Table 1:** Nutritional composition of the study drinks and oils.

	SUPP	CON
Whey protein (g)	30	0
Creatine (g)	2.5	0
Calcium (mg)	400	0
Vitamin D (IU)	500	0
Carbohydrate (g)	1	22
Energy (kcal)	116	56
	Oil	
EPA (mg)	1400	0
DHA (mg)	890	0

The quantities listed below represent a single drink and a single serving of oil. Participants consumed two drinks per day and one serving of oil per day.

SUPP, supplement; CON, control; EPA, eicosapentanoic acid; and DHA, docosahexaenoic acid.

### Exercise training

The 12 week progressive exercise training program took place at McMaster University. Subjects completed twice weekly RET (Mon and Fri) and once weekly HIIT (Wed).

The RET sessions began with a 5 min warm-up on a cycle ergometer, and thereafter subjects performed 3 sets of 4 separate exercises in the following order: leg press, chest press or lateral pull-down, horizontal row or shoulder press, and leg extension. Chest press and horizontal row were only performed on Monday, and lateral pull-down and shoulder press were only performed on Friday (leg press and leg extension were performed at every RET session). The RET sessions were concluded with a 5 min cool-down on a cycle ergometer. During the first 3 weeks of exercise training, workload was gradually increased from 65% 1RM (10–12 repetitions) to 80% 1RM (6–8 repetitions). The third set of each exercise was always completed until volitional fatigue, which we defined as the inability to complete an addition repetition with proper form. Loads were adjusted based on 1RM strength tests or when subjects could complete ≥ 12 repetitions during the third set of each exercise.

Participants performed HIIT on a cycle ergometer (ISO1000 Upright Bike; SCIFIT, Tulsa, OK) while wearing a heart rate (HR) monitor (H7 Heart Rate Sensor; Polar Electro Canada, Lachine, QC). Following a 3 min warm-up at 25 W, subjects completed 10 x 60 s intervals at a workload which elicited ~90% maximal HR (HRmax), while maintaining a cadence of ≥ 90 rpm. Workload was adjusted as needed to maintain an average HR of ~90% HRmax over the 10 intervals. Intervals were interspersed with 60 s of rest where subjects cycled at a self-selected pace against 25 W. HIIT sessions were concluded with a 5 min cool-down at 25 W.

### Strength assessments

At baseline, proper lifting technique was demonstrated and practiced by participants during a familiarization session. Muscle strength was assessed using 1RM strength tests for the following exercises: leg press, chest press, lateral pull-down, horizontal row, shoulder press, and leg extension (HUR; Northbrook, IL). The 1RM load was reassessed four days after the initial assessment as previously described [18]. Strength is reported as individual 1RMs for each of the six exercises, as well as the sum of upper body 1RMs (horizontal row, chest press, lateral pull-down, and shoulder press), the sum of lower body 1RMs (leg extension and leg press), and the sum of all 1RMs.

### Aerobic fitness testing

Subjects performed a VO_2_peak test on an electronically braked cycle ergometer (Lode Excalibur Sport V 2.0; Groningen, The Netherlands) while wearing a chest-strap HR monitor. A metabolic cart and online gas collection system (MOXUS Modular Oxygen Uptake System; AEI Technologies, Pittsburgh, PA) were used to quantify respiratory gases. Following a 1 min warm-up at 30 W, the load was increased by 1 W every 4 s. Participants were instructed to maintain a cadence of 60–90 rpm, and tests were terminated if the cadence dropped below 55 rpm for > 10 s, or if volitional fatigue was attained.

### Physical function

Subjects completed 3 assessments to measure physical function. The 30 s chair stand required subjects to rise from a chair without the use of their arms as many times as possible in 30 s [19]. For the timed up-and-go (TUG), subjects were instructed to rise from the same chair, walk to and from a clearly marked point a distance of 3 m away, and sit back down in the shortest amount of time possible. Subjects were given a practice trial before both the 30 s chair stand and the TUG, and the average of 3 trials (with 3 min rest allowed between trials) was recorded for each outcome. Lastly, the 6 min walk test was performed on a 200 m indoor track. Subjects were instructed to attempt to cover as much distance as possible within 6 min while walking in a safe manner at their usual walking speed.

### Body composition

Whole body and regional lean soft tissue mass (i.e. fat-free and bone-free mass), fat mass, and bone mineral content were measured using DXA (GE-LUNAR iDXA; GE, Mississauga, ON) following a 10–12 h overnight fast. Regional body compartment analysis was performed in batches by a single investigator who was blinded to group assignment. Waist and hip circumferences were measured at the top of the iliac crests and at the widest portion of the hips, respectively, using a tape measure while participants stood with their arms relaxed and feet together.

### Oral glucose tolerance test

After a 10h overnight fast, a 20 G catheter was inserted into an antecubital vein and a fasting blood sample (0 min) was obtained. Subjects then consumed a 75 g dextrose solution (Trutol™; NERL Diagnostics LLC, East Providence, RI) within 5 min. Serial blood samples were obtained at 30, 60, 90 and 120 min post-ingestion of the dextrose beverage for the measurement of plasma glucose and insulin concentrations. We calculated total (i,e. baseline values not subtracted) incremental area under the curve (AUC) for glucose and insulin using GraphPad Prism 7.0 (La Jolla, CA), according to previously described methods [20]. The Matsuda Index of insulin sensitivity was calculated using the equation formulated by Matsuda and Defronzo [21].

### Dietary intake

Three-day food records (2 weekdays and 1 weekend day) were analyzed using ESHA (Food Processor Nutrition Analysis Software; Salem, OR). Subjects were instructed by research staff on how to record the types and quantities of food, beverages, study drinks/oil, and other nutrition supplements or vitamins that they consumed during this period. Baseline 3-day food records were completed prior to commencing the study protocol.

### Biochemical analysis

Plasma glucose concentrations were measured using the glucose oxidase method (YSI 2300; Yellow Springs, OH). Plasma insulin concentrations were measured using the dual-site chemiluminescent method (Siemens Immulite 2000; Malvern, PA). Cholesterol (total, high-density lipoprotein [HDL], and low-density lipoprotein [LDL]) and triglycerides (TG) were analyzed using the Architect Clinical Chemistry Analyzer (Abbott Diagnostics; Lake Forest, IL). Plasma 25 hydroxyvitamin D (25[OH]D) concentrations were measured by radioimmunoassay using a commercially available kit (DiaSorin Canada; Mississauga, ON), and plasma cystatin C concentrations were measured with a BN100 Nephelometer (Dade Behring; Deerfield IL) using a particle-enhanced immunonephelometric assay.

Erythrocyte membrane phospholipid composition was measured as described previously [22]. Briefly, total lipids from the samples were extracted [23], and thin layer chromatography was used to separate individual classes of phospholipids (phosphatidylcholine, PC; phosphatidylethanolamine, PE; phosphatidylinositol, PI; phosphatidylserine, PS; and sphingomyelin, SM). Once isolated, phospholipids were methylated with 1 M methanolic sodium methoxide at room temperature for 10 min [24], and the fatty acid composition of each class of phospholipids was analyzed by gas chromatography (Hewlett-Packard 5890 Series II System, equipped with a double flame ionization detector, and Agilent CP-Sil 88 capillary column, 100 m, internal diameter of 0.25 mm) [25, 26]. Fatty acids were identified by comparing retention times to those of a known standard, and absolute amounts of individual fatty acids were calculated with the aid of an internal standard (pentadecanoic acid), which was added to samples before the methylation process. Total amounts of each phospholipid were determined from the sum of fatty acids in each fraction.

### Statistical analysis

Statistical analysis was completed using IBM SPSS software (IBM SPSS Statistics for Windows, version 23.0; IBM Corp., Armonk, NY). We conducted an intention-to-treat analysis using a linear mixed model with an unstructured covariance matrix including group (SUPP or CON), time (prior to study entry, and weeks 6 and 19), and the group-by-time interaction as fixed factors; subject as a random factor; and respective baseline values as covariates. Following significant group-by-time interactions, significant between (SUPP or CON) and within (weeks -1, 6, or 19) group differences were identified using post hoc tests with a Bonferroni correction for multiple comparisons. Based on recommendations for human clinical trials with missing data [27], all participants (completers as well as participants who withdrew prior to week 6 or week 19 testing) were included in the final analysis, and missing values were not replaced.

We also conducted an exploratory sub-analysis to examine whether baseline vitamin D status affected the amount of strength gained by participants in the SUPP group. For this sub-analysis we used a linear mixed model with an unstructured covariance matrix including subgroup (inadequate [< 50 nM] or adequate [≥ 50 nM] baseline plasma 25[OH]D, time (Phase 1 or 2), and the subgroup-by-time interaction as fixed factors; and subject as a random factor. Following significant subgroup-by-time interactions, significant between (inadequate or adequate baseline vitamin D status) and within (Phase 1 or 2) subgroup differences were identified using post hoc tests with a Bonferroni correction for multiple comparisons.

For all statistical analyses significance was accepted as *P* < 0.05. Data are presented as means ± standard error of the mean (SEM).

## Results

### Participants

Forty-nine older men were randomized: 38 completed the study and 11 dropped out (n = 7 and n = 4 dropouts in the SUPP and CON groups, respectively). Of the participants who dropped out, 4 withdrew prior to week 6 testing (during Phase 1), and 7 withdrew partway through the exercise training program and prior to week 19 testing (during Phase 2). Reasons for withdrawal from the study are provided in Fig 1. Participants’ baseline characteristics are presented in Table 2.

**Table 2:** Baseline characteristics of participants.

	SUPP (n = 25)	CON (n = 24)
Age (years)	71 ± 1	74 ± 1
Systolic BP (mm Hg)	138 ± 4	138 ± 3
Diastolic BP (mm Hg)	78 ± 2	78 ± 2
Body mass (kg)	85.3 ± 2.4	84.5 ± 2.5
Height (m)	1.72 ± 0.01	1.73 ± 0.01
BMI (kg/m^2^)	28.9 ± 0.8	28.1 ± 0.7
Whole body lean mass (kg)	54.0 ± 1.1	54.5 ± 1.4
Whole body fat mass (kg)	28.2 ± 1.7	26.8 ± 1.4
% body fat	33.6 ± 1.3	32.6 ± 1.0
Waist:hip ratio	0.99 ± 0.01	0.99 ± 0.01
Leg extension 1RM (kg)	27 ± 1	27 ± 2
Leg press 1RM (kg)	77 ± 3	69 ± 4
VO_2peak_ (mL/kg/min)	23.8 ± 0.8	24.4 ± 0.9
Peak power (W)	154 ± 5	158 ± 7
Fasting glucose (mM)	5.6 ± 0.1	5.8 ± 0.1
2h glucose (mM)	6.8 ± 0.4	7.2 ± 0.4
HOMA-IR	2.1 ± 0.1	2.2 ± 0.1
Total-c (mM)	4.69 ± 0.22	4.83 ± 0.19
LDL-c (mM)	2.74 ± 0.21	2.87 ± 0.18
HDL-c (mM)	1.27 ± 0.06	1.29 ± 0.06
TG (mM)	1.49 ± 0.19	1.50 ± 0.21
25(OH)D (nM)	44.3 ± 2.6	37.6 ± 2.8
Cystatin C (mg/L)	0.85 ± 0.03	0.83 ± 0.05

Data are means ± SEM. 25(OH)D, 25 hydroxyvitamin D; SUPP, supplement; CON, control; BP, blood pressure; BMI, body mass index; 1RM, one repetition maximum; HOMA-IR, homeostatic model assessment of insulin resistance; VO_2peak_, peak oxygen uptake; Total-c, total cholesterol; LDL, low-density lipoprotein; HDL, high-density lipoprotein; TG, triglycerides.

### Compliance

Compliance (based on self-report as well as returned sachets) with beverage consumption was: SUPP = 87 ± 2%; CON = 92 ± 2%. A similar degree of compliance was observed with oil consumption: SUPP = 92 ± 2%; CON = 95 ± 2%. Subjects in the SUPP and CON groups attended 95 ± 1% and 94 ± 1% of their prescribed training sessions, respectively. All subjects attended at least 80% of all RET and HIIT sessions.

### Blood

We assessed plasma 25(OH)D concentrations at weeks -1, 6, and 19. Concentrations of 25(OH)D were 44.3 ± 2.6 nM (SUPP) and 37.6 ± 2.8 nM (CON) at baseline (range: 21.0–64.2 nM; Table 2), and increased significantly in the SUPP group (to 50.5 ± 3.1 nM at week 6 and 57.1 ± 3.9 nM at week 19; *P* < 0.001). No change was observed in the CON group over the course of study (37.3 ± 2.6 nM at week 6 and 35.6 ± 2.5 nM at week 19; *P* > 0.05).

Erythrocyte membrane phospholipids were measured as described in detail previously [22], and the content of EPA plus DHA increased 90% (phosphatidylcholine), 22% (phosphatidylserine), 65% (phosphatidylinositol), and 43% (phosphatidylethanolamine) between baseline and week 6 in the SUPP group, and each increased an additional ~30% by week 19. The EPA plus DHA content of erythrocyte membranes did not change significantly over the course of the study in the CON group.

Cystatin C concentrations were 0.85 ± 0.03 mg/L (SUPP) and 0.83 ± 0.05 mg/L (CON) at baseline (range: 0.60–1.08 mg/L; see Table 2), and did not change significantly over the course of the study in either group.

### Isotonic muscle strength

**Fig 3:**
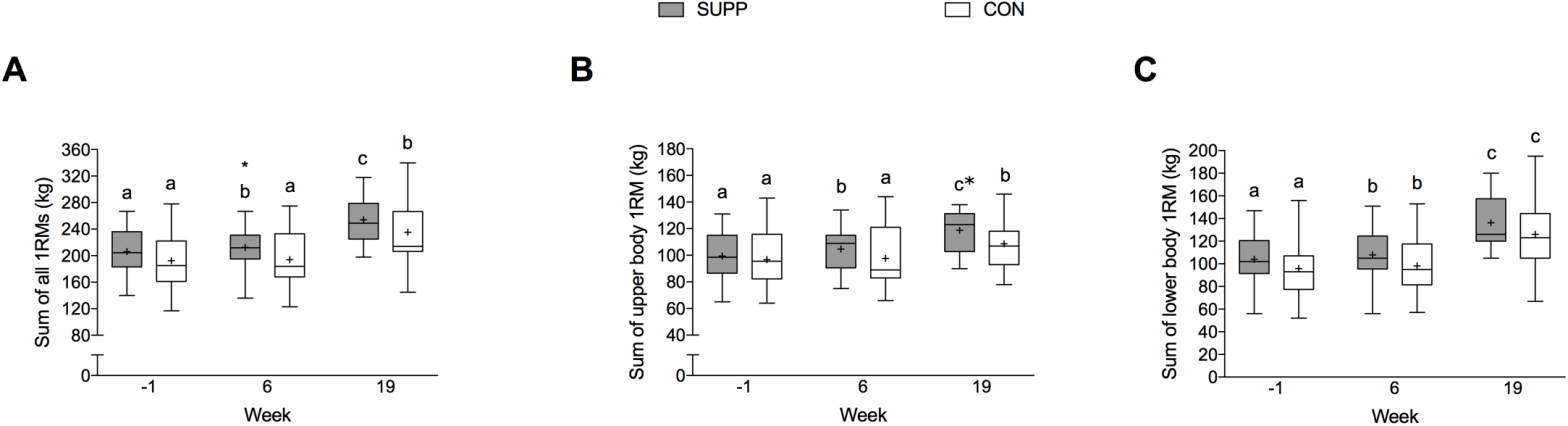
**Isotonic strength expressed as the sum of all (A), upper body (B), and lower body (C) 1RMs.** Boxes (SUPP: grey; CON: white) represent interquartile (25^th^ to 75^th^ percentile) ranges, with the horizontal lines indicating the median. Whiskers represent the maximal and minimal values, and the cross indicates the mean. Data were analyzed using a linear mixed model with group (SUPP or CON), time (prior to study entry, and weeks 6 and 19), and the group-by-time interaction as fixed factors; subject as a random factor; and baseline values as covariates. We observed significant group-by-time interactions for the sum of all 1RMs (*P* = 0.039) and for the sum of upper body 1RMs (*P* = 0.015), and a main effect of time for the sum of lower body 1RMs (*P* < 0.001). Dissimilar letters denote changes over time within a given treatment group (SUPP or CON). * Indicates a significant (*P* < 0.05) difference from the CON group at that time. 1RM, one repetition maximum; SUPP, supplement group (n = 25); CON, control group (n = 24).

Significant group-by-time interactions were observed for the sum of all 1RMs (*P* = 0.039) and upper body 1RMs (*P* = 0.015), as shown in Fig 3. In the SUPP group, the sum of all 1RMs increased by 3% during Phase 1 from 206 ± 7 kg at baseline to 212 ± 8 kg at week 6 (*P* < 0.001; Fig 3A) and a further 20% during Phase 2 (*P* < 0.001). In the CON group, there was no change in total (Fig 3A) or upper body (Fig 3B) strength during Phase 1. However, following Phase 2 the sum of all 1RMs increased by 21% (*P <* 0.001; Fig 3A) and the sum of upper body 1RMs increased by 11% (*P* < 0.001; Fig 3B) in the CON group.

### Body composition

**Fig 4:**
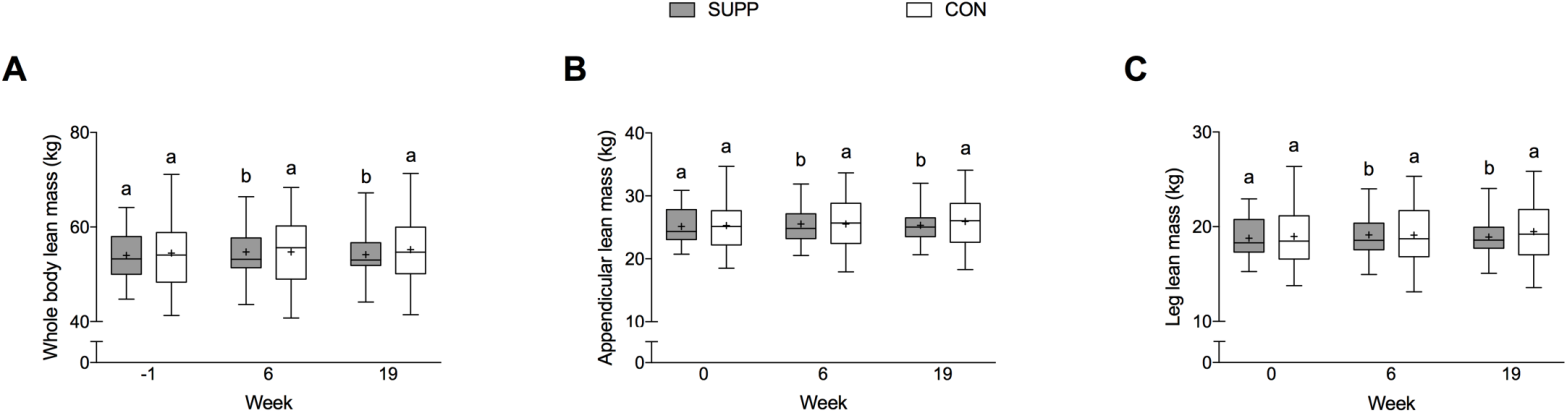
**Whole body (A), appendicular (B), and leg (C) lean mass over the course of the study.** Boxes (SUPP: grey; CON: white) represent interquartile (25^th^ to 75^th^ percentile) ranges, with the horizontal lines indicating the medians. Whiskers represent the maximal and minimal values, and the crosses indicate the means. Data was analyzed using a linear mixed model with group (SUPP or CON), time (prior to study entry, and weeks 6 and 19), and the group-by-time interaction as fixed factors; subject as a random factor; and baseline values as covariates. We observed significant group-by-time interactions for whole body (*P* < 0.01), appendicular (*P* = 0.021), and leg lean mass (*P* = 0.015). Dissimilar letters denote changes over time within a given treatment group (SUPP or CON). SUPP, supplement group (n = 25); CON, control group (n = 24).

We observed significant group-by-time interactions for whole body lean mass (*P* < 0.01), appendicular lean mass (*P* = 0.021), leg lean mass (*P* = 0.015), and trunk lean mass (*P* < 0.01). In the SUPP group, whole body lean mass increased by 0.7 kg in Phase 1 (*P* < 0.001; Fig 4A); however, no further increase was observed during Phase 2. Likewise, in the SUPP group at week 6 we observed increases in appendicular lean mass of 0.4 kg (*P* < 0.01; Fig 4B), leg lean mass of 0.3 kg (*P <* 0.01; Fig 4C), and trunk lean mass of 0.4 kg (*P* = 0.002; Table 3) compared to baseline. However, no further changes in appendicular, leg, or trunk lean mass were observed during Phase 2. In the CON group, conversely, we did not observe any significant change in whole body lean mass (Fig 4A) or regional measurements of lean mass over the course of the study.

**Table 3:** Anthropometric and DXA-derived body composition.

	SUPP			CON		
	Baseline	6wk	19wk	Baseline	6wk	19wk
Body mass (kg)^2^	85.3 ± 2.4^a^	85.9 ± 2.6^b^	83.1 ± 2.6^a,b^	84.5 ± 2.5^a^	85.3 ± 2.7^b^	85.9 ± 2.8^a,b^
BMI (kg/m^2^)^2^	28.9 ± 0.8^a^	29.2 ± 0.8^b^	28.9 ± 0.9^a,b^	28.1 ± 0.7^a^	28.4 ± 0.8^b^	28.5 ± 0.8^a,b^
Fat mass (kg)^2^	28.2 ± 1.7^a^	28.0 ± 1.9^b^	25.9 ± 2.0^b^	26.8 ± 1.4^a^	27.5 ± 1.5^b^	27.5 ± 1.6^a,b^
% body fat^2^	33.6 ± 1.3^a,b^	33.2 ± 1.4^a^	31.7 ± 1.6^b^	32.6 ± 1.0^a,b^	33.0 ± 1.1^a^	32.9 ± 1.1^b^
Arm lean mass (kg)^2^	6.3 ± 0.2^a^	6.4 ± 0.2^b^	6.4 ± 0.2^b^	6.3 ± 0.2^a^	6.4 ± 0.2^b^	6.5 ± 0.2^b^
Trunk lean mass (kg)^1^	25.1 ± 0.5^a^	25.5 ± 0.6^b^	25.2 ± 0.6^b^	25.5 ± 0.6^a^	25.4 ± 0.6^a^	25.5 ± 0.7^a^
Waist circumference (cm)	105 ± 2	105 ± 2	104 ± 2	104 ± 2	105 ± 2	104 ± 2
Hip circumference (cm)	106 ± 1	107 ± 1	107 ± 1	106 ± 1	106 ± 2	107 ± 2
Waist:hip ratio^2^	0.99 ± 0.01^a,c^	0.99 ± 0.01^b,c^	0.97 ± 0.01^a^	0.99 ± 0.01^a,c^	0.99 ± 0.01^b,c^	0.97 ± 0.01^a^

Values are means±SEM and were analyzed using a linear mixed model with group (SUPP or CON), time (prior to study entry, and weeks 6 and 19), and the group-by-time interaction as fixed factors; subject as a random factor; and baseline values as covariates. For each outcome, different superscript letters represent significant differences over time within each group. Significance accepted as *P* < 0.05. DXA, dual-energy x-ray absorptiometry; BMI, body mass index; SUPP, supplement group (n = 25); CON, control group (n = 24).

^1^Group-by-time interaction (*P* < 0.05).

^2^Main effect for time (*P* < 0.05).

Whole body fat mass increased significantly (*P* = 0.032) by ~0.2 kg during Phase 1 (SUPP: -1% and CON: +3%; Table 3) and subsequently decreased by 0.9 kg (*P* = 0.020) during Phase 2 (SUPP: -8% and CON: 0%).

### Vitamin D status sub-analysis

At baseline, 9/25 participants in the SUPP group had adequate vitamin D status. We observed a significant subgroup-by-time interaction for chest press and the sum of upper body 1RM strength (both *P* < 0.001). During Phase 1 participants in the SUPP group with inadequate and adequate baseline plasma 25(OH)D gained similar amounts of muscular strength. However during Phase 2, participants with inadequate baseline vitamin D status increased chest press (+4 ± 3 vs. +1 ± 4 kg, *P* = 0.041) and sum of upper body (+15 ± 7 vs. +7 ± 6 kg, *P* = 0.034) 1RM strength to greater extent compared to participants in the adequate subgroup.

### Physical function and aerobic fitness

We observed time-dependent increases in TUG, the 6 min walk test, VO_2_peak, and peak power (all *P <* 0.001; Table 4). There were no significant changes during Phase 1; however, significant improvements were made following Phase 2. Between weeks 6 and 19, the time taken to complete the TUG decreased by ~0.3 s (SUPP: -7% and CON: -3%; *P* = 0.004), and the distance covered in the 6 min walk test increased by ~25 m (*P* < 0.001). In addition, relative VO_2_peak increased overall by 1.8 mL/kg/min, and peak power increased by 13 W (*P* < 0.001). We did not observe any significant changes in the 30 s chair stand test over the course of study.

**Table 4:** Physical function and aerobic fitness assessments.

	SUPP			CON		
	Baseline	6wk	19wk	Baseline	6wk	19wk
30s chair stand (stands)^1^	12 ± 1^a^	13 ± 1^a,b^	13 ± 1^b^	13 ± 1^a^	13 ± 1^a,b^	13 ± 1^b^
Timed up-and-go (s)^1^	7.1 ± 0.2^a^	6.9 ± 0.3^a^	6.4 ± 0.2^b^	7.6 ± 0.3^a^	7.0 ± 0.3^a^	6.8 ± 0.3^b^
6 min walk (m)^1^	576 ± 14^a^	585 ± 15^a^	616 ± 19^b^	593 ± 17^a^	621 ± 16^a^	639 ± 23^b^
VO_2_peak (mL/kg/min)^1^	23.8 ± 0.8^a^	24.6 ± 0.9^a^	26.2 ± 1.2^b^	24.4 ± 0.9^a^	24.4 ± 1.1^a^	26.4 ± 1.4^b^
Peak power (W)^1^	154 ± 5^a^	157 ± 5^a^	164 ± 7^b^	158 ± 7^a^	158 ± 7^a^	178 ± 10^b^

Values are mean ± SEM and were analyzed using a linear mixed model with group (SUPP or CON), time (prior to study entry, and weeks 6 and 19), and the group-by-time interaction as fixed factors; subject as a random factor; and baseline values as covariates. For each outcome, different superscript letters represent significant differences over time within each group. Significance accepted as *P* < 0.05. SUPP, supplement group (n = 25); CON, control group (n = 24); TUG, timed up-and-go; VO_2_peak, peak oxygen uptake.

^1^Main effect for time (*P* < 0.05).

### Glucose tolerance

Glucose area under the curve (AUC, *P =* 0.002), insulin AUC (*P <* 0.001), the homeostatic model assessment of insulin resistance (HOMA-IR, *P <* 0.001), and the Matsuda index of insulin sensitivity (*P <* 0.001) all improved in a time-dependent manner following Phase 2 (Table 5). Interestingly, we noted a reduction in maximal glucose concentrations (Cmax) and mean glucose in the SUPP group that was progressive across the study, with no change in CON group.

**Table 5:** Fasting blood lipids and insulin sensitivity indices.

	SUPP			CON		
	Baseline	6wk	19wk	Baseline	6wk	19wk
Total-c (mM)^1^	4.69 ± 0.22^a^	4.34 ± 0.23^b^	4.56 ± 0.30^a,b^	4.83 ± 0.19^a,b^	4.86 ± 0.19^a^	4.73 ± 0.19^b^
LDL-c (mM)	2.74 ± 0.21	2.63 ± 0.19	2.77 ± 0.25	2.87 ± 0.18	2.87 ± 0.20	2.85 ± 0.19
HDL-c (mM)	1.27 ± 0.06	1.27 ± 0.08	1.28 ± 0.09	1.29 ± 0.06	1.29 ± 0.06	1.26 ± 0.07
TG (mM)^1^	1.49 ± 0.19^a^	0.97 ± 0.09^b^*	1.10 ± 0.14^b^	1.50 ± 0.21^a^	1.52 ± 0.18^a^	1.35 ± 0.16^a^
Glucose AUC (mM•120min)^2^	915 ± 16^a^	890 ± 10^a^	842 ± 14^b^	886 ± 11^a^	899 ± 12^a^	869 ± 13^b^
Mean plasma glucose (mM)^1^	7.2 ± 0.1^a^	7.0 ± 0.1^a,b^	6.7 ± 0.1^b^	7.0 ± 0.1^a^	7.1 ± 0.1^a^	6.9 ± 0.1^a^
Plasma glucose Cmax (mM)^1^	9.6 ± 0.3^a^	9.1 ± 0.2^a,b^	8.5 ± 0.2^b^	8.9 ± 0.2^a^	9.1 ± 0.2^a^	9.1 ± 0.2^a^
Insulin AUC (μIU/mL•120min)^2^	4080 ± 65^a^	3950 ± 77^a^	3533 ± 62^b^	4060 ± 69^a^	3951 ± 92^a^	3397 ± 68^b^
HOMA-IR^2^	2.1 ± 0.1^a^	2.1 ± 0.1^a^	1.7 ± 0.1^b^	2.2 ± 0.1^a^	2.0 ± 0.1^a^	1.7 ± 0.1^b^
Matsuda Index^2^	5.1 ± 0.7^a^	5.3 ± 0.6^a^	6.4 ± 0.8^b^	5.1 ± 0.6^a^	5.4 ± 0.8^a^	6.4 ± 0.8^b^

Values are mean ± SEM and were analyzed using a linear mixed model with group (SUPP or CON), time (prior to study entry, and weeks 6 and 19), and the group-by-time interaction as fixed factors; subject as a random factor; and baseline values as covariates. For each outcome, different superscript letters represent significant differences over time within each group. Significance accepted as *P* < 0.05. SUPP, supplement group (n = 25); CON, control group (n = 24); Total-c, total cholesterol; LDL, low-density lipoprotein; HDL, high-density lipoprotein; TG, triglyceride; Cmax, maximum concentration; AUC, area under the curve; HOMA-IR, homeostatic model assessment of insulin resistance.

^1^Group-by-time interaction (*P* < 0.05).

^2^Main effect for time (*P* < 0.01).

*Represents a significant difference from CON at that time point.

### Fasting lipids

We observed significant time-by-group interactions for TG (*P* = 0.023) and total cholesterol (*P* = 0.014), shown in Table 5. In the SUPP group, TG (-35%; *P* < 0.01) and total cholesterol (-7%; *P* = 0.003) were reduced following Phase 1 with no further reduction during Phase 2. In the CON group no change in TG was observed over the course of the study, however, total cholesterol decreased significantly (-3%; *P* = 0.026) following Phase 2.

### Dietary intake

We observed, as expected, significant group-by-time interactions for protein intake (expressed as g, g/kg body mass, and % energy; *P* < 0.001; Table 6), vitamin D (*P* < 0.001), calcium (*P* < 0.001), and n-3 PUFA (*P* = 0.003). In the CON group, we did not observe any significant change in macro- or micronutrient intake over the course of the intervention. A significant main effect of time was observed for daily energy intake (*P* = 0.004). Total energy intake was significantly higher at week 6 compared to baseline (SUPP: +12%; CON: +9%, *P* = 0.005), with no further change following Phase 2.

**Table 6:** Daily dietary intakes via self-report from 3-day food records.

		SUPP			CON		
		Baseline	6 wk	19 wk	Baseline	6 wk	19 wk
Energy (kcal)^2^		2146 ± 488^a^	2405 ± 701^b^	2375 ± 153^a,b^	2336 ± 553^a^	2541 ± 590^b^	2417 ± 757^a,b^
Protein							
	g^1^	89 ± 24^a^	142 ± 34^b^*	130 ± 25^b^*	97 ± 27^a^	100 ± 35^a^	99 ± 41^a^
	g/kg body mass^1^	1.1 ± 0.3^a^	1.7 ± 0.5^b^*	1.6 ± 0.4^b^*	1.2 ± 0.3^a^	1.2 ± 0.4^a^	1.2 ± 0.5^a^
	%^1^	17 ± 3^a^	24 ± 5^b^*	26 ± 5^b^*	17 ± 4^a^	16 ± 4^a^	17 ± 5^a^
Carbohydrate							
	G	265 ± 65	257 ± 87	223 ± 63	272 ± 102	309 ± 96	304 ± 121
	%^1^	50 ± 6^a^	43 ± 6^b^*	43 ± 6^b^*	46 ±12^a^	49 ± 10^a^	51 ± 13^a^
Fat							
	G	71 ± 22	82 ± 34	68 ± 32	86 ± 30	89 ± 35	80 ± 34
	%	30 ± 6	30 ± 6	28 ± 6	33 ± 8	31 ± 8	29 ± 8
Vitamin D (IU)^1^		161 ± 128^a^	1061 ± 220^b^*	1086 ± 168^b^*	175 ± 140^a^	193 ± 205^a^	148 ± 169^a^
Calcium (mg)^1^		775 ± 354^a^	1491 ± 617^b^*	1423 ± 305^b^*	944 ± 553^a^	919 ± 517^a^	856 ± 499^a^
n-3 PUFA (g)^1^		1.2 ± 1.4^a^	2.5 ± 1.4^b^*	2.3 ± 0.8^b^*	0.9 ± 0.7^a^	1.2 ± 1.0^a^	1.1 ± 1.3^a^

Values are mean ± SEM and were analyzed using a linear mixed model with group (SUPP or CON), time (prior to study entry, and weeks 6 and 19), and the group-by-time interaction as fixed factors; subject as a random factor; and baseline values as covariates. For each outcome, different superscript letters represent significant differences over time within each group. Significance accepted as *P* < 0.05. SUPP, supplement group (n = 25); CON, control group (n = 24); n-3 PUFA, omega-3 polyunsaturated fatty acids.

^1^Group-by-time interaction (*P* < 0.05).

^2^Main effect for time (*P* < 0.01).

*Represents a significant difference from CON at that time point.

## Discussion

We report that twice daily consumption of a whey protein-based, multi-ingredient supplement resulted in significant gains in muscle strength and lean mass. In addition, muscle strength, physical function, aerobic capacity and metabolic health were further improved in response to a 12 week combined exercise training program. Some, but not all, outcomes were improved to a greater extent with supplementation. While it is not possible to isolate which compounds in the supplement were responsible for the outcomes observed, each ingredient has been shown to independently affect aspects of sarcopenia and thus has a rational basis for inclusion. Notably, whey protein supplementation can variably enhance lean mass and strength [4, 28–31], creatine can improve strength [5, 32] vitamin D supplementation can reduce the risk of falls [33] and fractures [34], and n-3 PUFA has been shown it to improve muscle quality [35], mass [11], and function [11, 35] in older adults. However, the response heterogeneity for all relevant variables to the individual compounds provides a rationale for why a combination of ingredients could be more efficacious than any of the compounds alone. We excluded women from this relatively small trial in an attempt to reduce the variability in strength and lean body mass measurements. Given that women make up a large proportion of older adults and may be more vulnerable than men to anabolic resistance [36], we acknowledge this as a major limitation.

Some studies [11, 37, 38] have demonstrated a beneficial effect of nutritional supplementation independent of exercise on muscle strength, physical function, and lean mass in older adults, but others have not [39, 40]. This discrepancy may, in part, be explained by a heterogeneity of response to supplementation in older adults. By employing a multi-ingredient approach to dietary supplementation we hypothesized that we would be more likely to influence more than one potentially important pathway/mechanism through which older persons would derive an anti-sarcopenic effect. The present study clearly showed increases in total muscle strength and lean mass in response to a multi-ingredient nutritional supplement in the absence of exercise training. Other trials using a combination of ingredients (whey protein [leucine] and vitamin D) similar in dose to those we employed in our supplement have shown preservation of lean mass during weight loss [15], enhanced strength [16], increased lean body mass [16], and enhanced physical function (in sarcopenic older persons) [16]. Such changes in lean body mass and strength are clinically relevant given that strength and muscle mass decrease at the respective rates of ~1–3% and ~0.5–1% annually, likely commencing in or around the fifth decade of life [41]. Notably, the gains in strength and lean body mass we observed would be the equivalent of offsetting about one year of age-related decline in these variables, suggesting that this formulation, especially when combined with exercise, could mitigate the progression of sarcopenia.

Exercise training is another highly effective intervention to counter sarcopenia. Resistance exercise, in particular, acutely elevates myofibrillar protein synthesis for several days [12], resulting in long-term gains in muscle mass and strength in older adults when practiced regularly [42, 43]. Previously, we have shown that a single bout of high intensity interval exercise stimulates myofibrillar protein synthesis in older adults [12]. High intensity interval exercise offers numerous other health benefits in both younger and older populations, such as improvements in glycemic regulation [13] and aerobic capacity [14]. Knowing that aging is associated with reduced cardiometabolic health, as well as low muscularity and strength, we designed an exercise training program that combined exercise modalities for optimal benefit in older adults. Our data show that RET and HIIT can be safely used in combination to elicit significant gains in lean mass, strength, and physical function. Additionally, markers of cardiometabolic health, such as aerobic capacity and glucose tolerance, also improved in this sample of older men (Table 5). The lack of a significant increase in lean mass during Phase 2 is likely due to the relatively low volume of resistance exercise performed by participants in the current study compared to previous studies [44]. It is also possible that there was a potential antagonizing effect of concurrent exercise (i.e., HIIT) on RET-induced adaptations [45]. Nonetheless, the significant gains in muscle strength and function that we observed following exercise training may be important for physical function, mobility, and a higher quality of life [46].

We show that consumption of the multi-ingredient supplement during the exercise training program (Phase 2) resulted in superior gains in upper body strength compared to CON; however, we did not observe the same findings in lower body or overall muscle strength (Fig 3), lean mass (Fig 4), or physical function (Table 4). Several previous studies have failed to identify an additional beneficial effect of nutritional supplementation on exercise-mediated gains in lean mass and strength [35, 47–49]. Conversely, other studies have reported a positive effect [50–52]. Sample size may have limited our ability to detect a difference between the supplement and control group over the course of exercise training. A post hoc calculation revealed that we would have required 80 participants per group to detect a difference in the magnitude of overall strength. The relative good health of the participants included in the present study also may have prevented us from observing such an effect. We postulate that if the same exercise and nutrition intervention were applied to a more frail/functionally impaired group of older adults [16], it is possible that the same multi-ingredient supplement would be even more efficacious.

The current results demonstrate that this multi-ingredient supplement may be beneficial in patients for whom structured exercise is not possible or who are undergoing periods of muscle disuse. This is of particular importance since the relatively slow and steady decline in strength and muscle mass with age is often punctuated by brief periods of muscle disuse (i.e., during hospitalization) where losses are accelerated [53, 54]. While we cannot isolate the effects of specific nutrients some associations we observed are, we propose, noteworthy. In an exploratory subgroup analysis, we observed that a transition from insufficient vitamin D status (serum 25[OH]D < 50 nM) at baseline to sufficient positively influenced the degree of strength gained by participants in the SUPP group. Given that vitamin D status and protein intake increased significantly with nutritional supplementation over the course of this study, these observations are consistent with previous studies that report an association between strength [55] and lean body mass [56–58] gains. The results of this subgroup analysis therefore reinforce the potential of multi-ingredient nutritional supplementation to target several nutritional requirements simultaneously, increasing its applicability to a wider range of older adults.

In conclusion, we have demonstrated that twice daily consumption of a whey protein-based supplement containing creatine, vitamin D, calcium, and n-3 PUFA was effective in stimulating strength and lean body mass gains in the absence of exercise in a group of healthy older men. Future trials with a larger sample size, and including older women, for a greater duration would confirm if this supplement represents a viable anti-sarcopenic strategy with the potential for broader use.

## Supporting information

S1 File Study protocol. The approved study protocol.
(DOCX)

S2 File CONSORT study checklist. (DOCX)
