# Correction: A whey protein-based multi-ingredient nutritional supplement stimulates gains in lean body mass and strength in healthy older men: A randomized controlled trial

**DOI:** 10.1371/journal.pone.0243876

**Published:** 2020-12-15

**Authors:** Kirsten E. Bell, Tim Snijders, Michael Zulyniak, Dinesh Kumbhare, Gianni Parise, Adrian Chabowski, Stuart M. Phillips

The following information is missing from the Competing Interests statement: A preliminary patent application was filed by McMaster University on behalf of SMP and GP prior to publication. The rights to this patent are now assigned to Exerkine Corporation for the multi-nutrient supplement used in this study. This patent is now published as International Patent No. WO/2018/157258, September 7, 2018.

Additionally, the Data Availability statement for this paper is incorrect. The correct statement is: Data underlying the study are available on the OSF repository at DOI: 10.17605/OSF.IO/H35QC.
