# Supplementary material for: A whey protein-based multi-ingredient nutritional supplement stimulates gains in lean body mass and strength in healthy older men: A randomized controlled trial
Source: PLoS One. 2017 Jul 18;12(7):e0181387. doi: 10.1371/journal.pone.0181387 (PMC5515445; doi:10.1371/journal.pone.0181387)
Supplement: S1 File — The approved study protocol. (DOCX) [file pone.0181387.s001.docx]

**Research Project Proposal**

**Title: Evaluation of the effects of a combined resistance and high-intensity interval exercise training program, along with nutritional supplementation, on strength and physical function in older men**

**Principle Investigator: Gianni Parise, Ph.D.**

**Co-Investigators: Stuart Phillips, Ph.D.**

**Jennifer Heisz, Ph.D.**

**Tim Snijders, Ph.D.**

**Kirsten Bell, M.Sc.**

**Dinesh Kumbhare, M.D.**

**1.0 Rationale**

Currently in Canada, trends indicate that there is an increasing number of aging Canadians with longer life spans^1^. Thus, there is a greater need to identify and understand the underlying physiological mechanisms of the aging process. One area of interest revolves around understanding the significant decline in lean muscle mass and strength as we age. These phenomena are referred to as sarcopenia and dynapenia, respectively, and can have profound negative effects on muscle function and overall health. Sarcopenia and dynapenia accelerate the progression of various chronic diseases such as type 2 diabetes and cardiovascular disease, and lead to an increased incidence of injuries^2^. Consequently, the search for the best therapeutic strategy to counterbalance the loss of muscle mass and strength is ongoing.

Aged muscle, similarly to young muscle, retains the ability to positively respond to a physiological stimulus such as resistance exercise (RE)^3^. The result is that older adults who lift weights are capable of increasing both their strength and muscle mass, facilitating their ability to perform day-to-day activities, such as lifting heavy grocery bags and climbing stairs. Thus, continued regular exercise is currently the best therapeutic strategy to improve mobility and counteract the loss of muscle. Recently, our group demonstrated that similarly to RE, a bout of high-intensity interval exercise (HIIT) was able to generate a significant anabolic response in a group of older men. Namely, HIIT significantly stimulated muscle protein synthesis (MPS), as well as elicited a satellite cell response up to 48 hours post-exercise (unpublished results). These observations lead us to speculate that a training program combining HIIT, which consists of repeated bouts of vigorous aerobic exercise interspersed with period of rest, and RE might induce favourable changes in body composition and physical function in older men.

Despite the responsiveness of older muscle exercise, studies suggest that older muscle requires greater nutritional support compared to younger muscle. For instance, older men require ~40g whey protein to maximally stimulate MPS, whereas younger men only require ~20g^4^. This nutritional ‘anabolic resistance’ of older muscle can be alleviated with dietary strategies. Increased protein intake, particularly when combined with exercise training, is effective in bolstering muscle mass^5^. Further, creatine^6^, calcium^7^ and vitamin D^8^ have been shown to independently increase strength in older men, and omega-3 fatty acids have been shown to increase rates of MPS in older adults^9^ It is possible that a protein-based supplement that contains creatine, calcium, vitamin D and omega-3 fatty acids may exert an additive or even synergistic effect on muscle mass and strength, particularly if combined with a progressive exercise training program. To date, however, no one has tested this hypothesis.

**2.0 Objectives**

This study will examine the effects of a 12 week RE + HIIT exercise training program combined with multi-ingredient protein-based supplementation (SUPP), compared to 12 weeks of training combined with placebo (PLB), in older men. The substantive objectives of this study are to determine:

1. Primarily, whether the supplement is able to enhance exercise-mediated changes in muscle strength, physical function and body composition.
2. The acute satellite cell response to a single bout of HIIT + RE following 12 wks of protein or placebo supplementation.
3. The effect of each intervention on:
   1. Glucose metabolism and fasting serum lipids.
   2. Cognitive and executive function
   3. Renal function.

**3.0 Hypotheses**

1. Supplementation and exercise training will independently and synergistically increase muscle strength and physical function, as well as augment fat-free mass (FFM) and reduce fat mass (FM).
2. The acute satellite cell response will be greater/less impaired after 12 weeks of protein supplementation compared with placebo.
3. Myofibrillar and sarcoplasmic MPS, glycemic control, lipid partitioning, executive function and quality of life will improve in response to supplementation + exercise and exercise alone, and to a lesser extent following supplementation alone. Neither supplementation nor placebo training will alter renal function.

**4.0 Population under study**

For this study, we will recruit 50 sedentary yet healthy men aged 70 years or older (n=25 per group). Participants will be considered sedentary if they have not participated in a structured resistance or aerobic training program in the past 6 months. In addition, participants will have a BMI in the normal or overweight range (18.5 – 30.0 kg/m^2^), will have been weight stable for the past 6 months, and will present with a normal resting blood pressure or stage I hypertension at screening (SBP < 140 – 159 mmHg; DBP < 90 – 99 mmHg).

The following exclusion criteria exist to ensure the safety of participants and that certain outcome measures are not confounded:

- Smoking
- Use of simvastatin or atorvastatin
- Use of non-steroidal anti-inflammatory drugs
- Injuries that prevent safe participation in an exercise program
- Prescription blood-thinners
- Diabetes or other metabolic disorders
- Cardiac or gastro-intestinal problems
- Infectious disease or cancer

*4.1 Screening*

A variety of questionnaires and physiological tests will be used to assess eligibility for the study:

Medical Screening Questionnaire and Physical Activity Readiness Questionnaire (PAR-Q): Members of the research team will help potential participants complete a health questionnaire, which will assess the potential participant’s age, physical activity levels, and any medications and medical conditions that the individual may have. We will also measure height and weight. Potential participants must answer ‘no’ to all questions on the PARQ.

OGTT: Potential participants will also undergo a 2 hour 75g OGTT to determine whether they are diabetic (fasting blood glucose > 7.0 mM; 2 hour blood glucose > 11.1 mM) or display impaired glucose tolerance (fasting blood glucose > 5.6 mM; 2 hour blood glucose > 7.8 mM). Details of the OGTT protocol are described below.

Blood pressure: Systolic and diastolic blood pressure must be within the range indicated above in order for an individual to participate in this study. Participants will quietly rest in the supine position for 5 min before measurements are made using an automatic blood pressure cuff. A total of 3 measurements will be made, and the average will be used to assess resting blood pressure.

Exercise stress test: Participants must demonstrate normal cardiac function during a graded, maximal exercise stress test on a cycle ergometer using a 12-lead ECG. All exercise stress tests will be conducted at the 3U Cardiorespiratory Clinic at McMaster University Medical Centre, under the supervision of a trained technician.

**5.0 Sample size**

A total of 50 old, active men will be recruited; participants will be separated into 2 groups of 25 and each group will receive either SUPP or PLB. Previous studies have shown that supplementation with creatine monohydrate during RE training in older adults can increase dynamic strength by approximately 1.75 – 3.25 kg more than RE training alone. Using this effect size and a standard deviation of 1.5 kg, n = 20 subjects are needed to detect a difference with α = 0.05 and β = 0.8 using a two-way ANOVA. To account for the possibility of increased variability in the sample set and allowing for a potential 20% dropout rate, a conservative number of 25 subjects per group was chosen.

**6.0 Design**

The overall study design is depicted in Figure 1, below. Participants will be placed in either the SUPP or PLB group, depending on their strength, fat-free mass and body mass index.


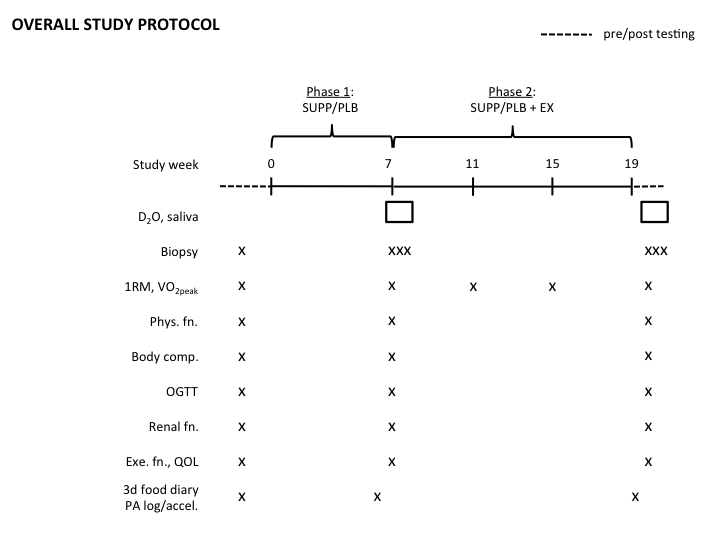


**Figure 1**. Study schematic. D_2_O, deuterium oxide; 1RM, 1 repetition maximum; VO_2peak_, peak oxygen consumption; OGTT, oral glucose tolerance test; QOL, quality of life assessment, PA, physical activity

*6.1 Assessments*

Approximately 1 week prior to beginning the study, participants will receive a muscle biopsy from *vastus lateralis*, exercise and physical function testing, body composition analysis, an OGTT, and an executive function and quality of life assessment (see Figure 1). These tests will take place over the course of 3-5 days during the same week. During the week these assessments are taking place, participants will complete a 3-day food diary and physical activity log at home, as well as wear an accelerometer (mounted on an arm band) to estimate daily caloric intake, macronutrient distribution and energy expenditure. We will assess glomerular filtration rate (GFR), an estimate of kidney function, by examining cystatin C concentrations in blood samples collected during pre, mid and post-study testing periods.

These assessments will be repeated during week 7, and again upon completion of the study during week 19. Exercise tests (1RM and VO_2peak_) will be repeated at weeks 11 and 15 to ensure an adequate exercise stimulus is maintained during training. The assessments at week 7 and week 19 include serial muscle biopsies for the measurement of the acute satellite cell response to exercise. Details of the serial muscle biopsy protocol are described below. Participants will be asked to refrain from strenuous exercise, caffeine and alcohol for 48 hours prior to each assessment.

*6.2 Phase 1*

Phase 1 of the study is 6 weeks in duration and will be completed primarily at home. Participants will consume 2 study beverages per day – the first approximately 1 hour after breakfast, and the second before bed. Participants will be encouraged to maintain their normal diets and levels of physical activity. We will provide participants with all their study beverages at the beginning of the study.

*6.3 Phase 2*

During Phase 2, participants will perform 3 exercise sessions per week for 12 weeks, while continuing to consume 2 study beverages per day. On non-exercise days, they will consume their beverages exactly as they did in Phase 1: the first 1 hour after breakfast and the second before bed. However on days when they visit McMaster University to exercise, participants’ first study drink of the day will be provided to them upon completion of the exercise session. The second study drink will be taken before bed, as usual.

*6.4 Exercise training*

Exercise training will take place three times per week for 12 weeks. One session per week will consist of HIIT on a bicycle ergometer, and 2 sessions per week will consist of whole body RE. Before beginning a HIIT session, participants will warm up on the bicycle ergometer for 5 min at a low resistance. The exercise will consist of 10 bouts of 60s where participants will sprint (ie. maintain a cadence of at least 90 rpm) at a power predetermined to elicit 90% HR_max_. These bouts will be interspersed with 60s of ‘rest’, where participants will peddle at ~25W at a minimum cadence of 60 rpm. Participants will wear a HR monitor during each session to ensure they are exercise at a safe, appropriate intensity. All HIIT sessions will be followed by a 5 min cool-down at ~25W.

During each whole body RE exercise session, participants will complete 2 upper body and 2 lower body exercises. Leg extension and leg press will be completed during every session, however the upper body exercises will alternate between chest press/horizontal row and shoulder press/biceps curl. For each exercise, participants will complete 3 sets of 8-12 repetitions at 80% 1RM, with the last set completed to failure. Each exercise session will be supervised by a member of the research team.

*6.5 Acute exercise protocol*

To assess the integrated MPS and acute satellite cell response to SUPP in the untrained and trained state, a set of 3 muscle biopsies (1 per day for 3 days) will be conducted at weeks 7 and 19. Immediately prior to participants’ first exercise session in week 7, a muscle biopsy will be taken (time-point: 0 hours). Biopsies will be taken again at 24 and 48 hours post-exercise. This process will be repeated ~1 week following participants’ last exercise session in week 19. Approximately ~ 8 mL of blood will be drawn from an antecubital vein after each biopsy is taken.

*6.6 Deuterated water protocol*

Immediately after the 0 hour muscle biopsies at weeks 7 and 19, but before completing their exercise session, participants will consume a bolus dose of D_2_O (150 mL). Saliva samples will be taken in the morning each day that the participant receives a muscle biopsy. These saliva samples can be taken by participants at home using a simple salivette, and must be taken prior to eating, brushing teeth and consuming D_2_O.

**7.0 Measurements**

*7.1 Strength and aerobic fitness*

Muscle strength will be assessed using 1 repetition maximum (1RM) testing of the following exercises: leg extension, leg press, chest press, horizontal row, lat pulldown and shoulder press. A 1RM test measures the maximum amount of weight someone can lift for a single repetition, while maintaining good technique. Aerobic fitness (VO_2peak_) will be measured using a graded, maximal exercise test on a bicycle ergometer. Briefly, participants will wear a facemask and begin peddling at ~50-70 rpm at a low resistance. The resistance will gradually increase until participants are no longer able to maintain a cadence of 50 rpm or until they request to stop.

*7.2 Physical function*

The short physical performance battery (SPPB) tests, along with grip strength and a 6 min walk test, will be used to assess physical function. The SPPB tests include a repeated chair stand test (time it takes for participants to stand up from a chair 5 times), a balance test and 8 feet walk test. The scoring system for the SPPB is standardized and will allow us to compare our results to the literature.

*7.3 Body composition*

FM, FFM and bone mineral density will be measured using dual-energy x-ray absorptiometry (DXA), and muscle thickness of the quadriceps muscle will be determined using ultrasound. Waist circumference will be measured using a tape measure at the top of the iliac crest.

*7.4 Satellite cell response*

Muscle cross-sections will be mounted onto glass slides and histochemically analyzed for the following markers of satellite cell activity: Pax7, MyoD, myogenin, myostatin, IL-6, GDF11.

*7.5 Muscle protein synthesis*

We will determine myofibrillar and sarcoplasmic fractional synthetic rate (FSR) by measuring the incorporation of ^2^H labeled alanine into muscle proteins using the precursor-product method. Following consumption of a 150 mL dose of D_2_O, ^2^H will quickly equilibrate with total body water (precursor pool). ^2^H will also become incorporated into the amino acid alanine, which in turn will become incorporated in muscle proteins. Using saliva swabs to assess ^2^H in the precursor pool, and muscle biopsies to assess ^2^H in the product pool, we can measure FSR in various muscle subfractions. Muscle samples will be purified in our laboratory. Both muscle and saliva samples will be analyzed using gas chromatography pyrolysis isotope ratio mass spectrometry (GC-*pyrolysis*-IRMS) and cavity ring-down spectrometry, respectively.

*7.6 Glucose metabolism*

A 2 hour 75g OGTT will be used to assess aspects of glucose metabolism. Briefly, after an overnight fast (no food or drink except for water after midnight the night before) a catheter will be inserted into an antecubital vein and a fasting blood sample will be drawn (~16 mL). Participants will then consume a beverage that contains 75g glucose within 5 minutes. Blood (~8 mL) will be drawn at the following time-points: 15 min, 30 min, 45 min, 60 min, 90 min and 120 min. The total amount drawn will be ~64 mL. We will measure blood glucose and insulin concentrations at every time-point, as well as the area under the glucose and insulin curves.

Other variables will also be measured in the fasting blood sample:

- Lipids: total cholesterol (TC), low-density lipoprotein cholesterol (LDL-c) and high-density lipoprotein cholesterol (HDL-c), as well as triacylglycerol (TAG) concentrations.
- Markers of bone health: 25-hydroxyvitamin D (25[OH]D), parathyroid hormone (PTH), osteoprotegerin (OPG), receptor activator of nuclear factor kappa-B ligand (RANKL)
- Liver enzymes and proteins: alanine aminotransferase (ALT), aspartate aminotransferase (AST), gamma glutamyl transpeptidase (GGT), albumin and total protein
- Cystatin C (for the estimation of glomerular filtration rate [to assess renal function])

*7.7 Cognitive function and quality of life*

Cognitive performance will be evaluated using validated neuropsychological assessment tools and cognitive tests will be included to get a full range of cognitive function:

| **Area of Interest** | **Validated Assessment Tools** |
| --- | --- |
| Global cognitive ability | - Montreal Cognitive Assessment (MoCA)^10^ - National Adult Reading Test (NART)^11^ |
| Language | - Boston Naming Test (BNT)^12^ - Controlled Oral Word Association Test^13^ |
| Memory | - Face recognition memory paradigm^14,15^ - Hopkins Verbal Learning Test –Revised (HVLT-R)^16^ - Brief Visual Memory Test - Revised (BVMT-R)^17^ - Rey Complex Figure Test, Wechsler Memory Scale – Revised - Logical Memory subtest, Autobiographical Interview – Abbreviated Administration^18^ |
| Attention | - Wechsler Adult Intelligence Scale – 3^rd^ Edition (WAIS-III) Digit Span Subtest^19^ |
| Inhibitory control | - Erikson Flanker Task^20^ |
| Working memory | - Directed Forgetting Test^21^ |

We will evaluate quality of life with a series of questionnaires designed to explore sleep quality, stress levels, daily activities, geriatric depression and subjective memory.

*7.8 Renal function*

We will estimate GFR using serum cystatin C concentrations. This will be measured using the fasting blood samples collected during the OGTTs performed at pre, mid (week 7) and post-study (week 19) testing.

*7.9 Diet analysis and physical activity*

Participants will complete a 3-day food diary and PA log during each assessment week. They will record everything the eat and drink, as well as any exercise they engage in, for 2 week days and 1 weekend day. Additionally, participants will wear and accelerometer (mounted on an armband) for the same 3 days so that we may estimate daily energy expenditure. The 3-day food diary, PA log and accelerometer will allow us to approximate daily caloric intake and macronutrient distribution, as well as to determine whether their dietary and activity patterns have changed throughout the course of the study.

**8.0 Risks**

The potential risks and discomforts associated with the exercise testing and training procedures are similar to those associated with any form of strenuous physical activity. These include fatigue, fainting, abnormal blood pressure, irregular heart rhythm, and in very rare instances, heart attack, stroke or death. Every effort will be made to minimize these potential risks by ensuring all participants receive an exercise stress test prior to beginning the study, by evaluating preliminary information related to the health and fitness levels of subjects (using the health questionnaire and PAR-Q), and by careful observations during testing and training. Should any acute injury occur or if further medical assistance becomes required, we shall ensure that the necessary care is provided to our volunteers.

There is a risk of infection, bruising and soreness any time the skin is breached, as with muscle biopsies and blood sampling. To minimize risk and discomfort to our participants, all biopsies will be performed by Tim Snijders, Ph.D., who has training and experience with this procedure. All blood draws will be conducted by trained members of the research team, and all equipment will be sterile. Participants will be instructed on the proper care of their biopsy blood draw sites.

The risk associated with DXA and D_2_O is minor. DXA involves the use of whole-body x-ray, however the dose of radiation is less than one would receive when taking a transatlantic flight. Nausea and vertigo have been reported as side-effects of D_2_O consumption. However, the dose used in this study is small and unlikely to cause any symptoms.

**9.0 Data analysis**

Data will be analyzed using a 2-way analysis of variance (ANOVA) with between (SUPP, PLB) and within (time) group comparisons. Tukey’s HSD post hoc testing will be used to assess differences between the means when interactions are detected. Statistical significance will be accepted at α<0.05.

**10.0 Recruitment**

Participants will be recruited from the Hamilton and surrounding area through the posting of study advertisement posters in newspapers and on the McMaster Campus (see Appendix 4). It is anticipated that all subjects will be unknown to the investigators. All subjects will review the consent form prior to receiving a signed copy. Participants will be encouraged to contact any of the investigators if they have any questions or concerns about the study. A copy of the signed consent form will also be added to the participants’ data file.

**11.0 References**

1. Milan, A. Age and sex structure: Canada, provinces and territories, 2010. *Statistics Canada* 1–9 (2011).

2. Yu, R., Leung, J. & Woo, J. Incremental predictive value of sarcopenia for incident fracture in an elderly Chinese cohort: results from the osteoporotic fractures in men (MrOs) study. *J Am Med Dir Assoc* **15,** 551–558 (2014).

3. Volpi, E., Sheffield-Moore, M., Rasmussen, B. B. & Wolfe, R. R. Basal muscle amino acid kinetics and protein synthesis in healthy young and older men. *JAMA* **286,** 1206–1212 (2001).

4. Yang, Y. *et al.* Resistance exercise enhances myofibrillar protein synthesis with graded intakes of whey protein in older men. *Br J Nutr* **108,** 1780–1788 (2012).

5. Chale, A. *et al.* Efficacy of Whey Protein Supplementation on Resistance Exercise-Induced Changes in Lean Mass, Muscle Strength, and Physical Function in Mobility-Limited Older Adults. *J. Gerontol. A Biol. Sci. Med. Sci.* **68,** 682–690 (2013).

6. DEVRIES, M. C. & Phillips, S. M. Creatine Supplementation during Resistance Training in Older Adults—A Meta-analysis. *Medicine & Science in Sports & Exercise* **46,** 1194–1203 (2014).

7. Hidalgo, J. L.-T. & Group, A. Prevention of falls and fractures in old peopleby administration of calcium and vitamin D. *BMC Public Health* **11,** 910 (2011).

8. Rizzoli, R. *et al.* ARTICLE IN PRESS. *Maturitas* 1–11 (2014). doi:10.1016/j.maturitas.2014.07.005

9. Smith, G. I. *et al.* Dietary omega-3 fatty acid supplementation increases the rate of muscle protein synthesis in older adults: a randomized controlled trial. *American Journal of Clinical Nutrition* **93,** 402–412 (2011).

10. Nasreddine, Z. S. *et al.* The Montreal Cognitive Assessment, MoCA: a brief screening tool for mild cognitive impairment. *Journal of the American Geriatrics Society* **53,** 695–699 (2005).

11. Nelson, H. E. *The National Adult Reading Test (NART): Test Manual*. (NFER-Nelson, 1982).

12. Borod, J. C., Goodglass, H. & Kaplan, E. Normative data on the Boston Diagnostic Aphasia Examination, parietal lobe battery, and the Boston Naming Test. *Journal of Clinical and Experimental Neuropsychology* **2,** 209–213 (1980).

13. Ruff, R., Light, R., Parker, S. & Levin, H. Benton controlled oral word association test: Reliability and updated norms. *Archives of Clinical Neuropsychology* **11,** 329–338 (1996).

14. Heisz, J. J. & Shore, D. I. More efficient scanning for familiar faces. *Journal of Vision* **8,** 9.1–10 (2008).

15. Heisz, J. J. & Ryan, J. D. The effects of prior exposure on face processing in younger and older adults. *Frontiers in Aging Neuroscience* **3,** 15 (2011).

16. Benedict, R. H., Schretlen, D., Groninger, L. & Brandt, J. Hopkins Verbal Learning Test–Revised: normative data and analysis of inter-form and test-retest reliability. *The Clinical Neuropsychologist* **12,** 43–55 (1998).

17. Benedict, R. H., Schretlen, D., Groninger, L., Dobraski, M. & Shpritz, B. Revision of the Brief Visuospatial Memory Test: studies of normal performance, reliability, and validity. *Psychological Assessment* **8,** 145 (1996).

18. Levine, B., Svoboda, E., Hay, J. F., Winocur, G. & Moscovitch, M. Aging and autobiographical memory: Dissociating episodic from semantic retrieval. *Psychology and Aging* **17,** 677–689 (2002).

19. Axelrod, B. N., Fichtenberg, N. L., Millis, S. R. & Wertheimer, J. C. Detecting Incomplete Effort with Digit Span from the Wechsler Adult Intelligence Scale—Third Edition. *The Clinical Neuropsychologist* **20,** 513–523 (2006).

20. Eriksen, C. W. The flankers task and response competition: A useful tool for investigating a variety of cognitive problems. *Visual Cognition* **2,** 101–118 (1995).

21. Zacks, R. T., Radvansky, G. & Hasher, L. Studies of directed forgetting in older adults. *Journal of Experimental Psychology: Learning, Memory and Cognition* **22,** 143 (1996).
